# Minimally invasive lateral retroperitoneal transpsoas approach for lumbar corpectomy and fusion with posterior instrumentation

**DOI:** 10.3171/2022.3.FOCVID2210

**Published:** 2022-07-01

**Authors:** Ethan S. Srinivasan, Timothy Y. Wang, Anna Rapoport, Melissa M. Erickson, Muhammad M. Abd-El-Barr, Christopher I. Shaffrey, Khoi D. Than

**Affiliations:** 1 Duke University School of Medicine;; 2 Department of Neurosurgery, Duke University Medical Center; and; 3 Department of Orthopaedic Surgery, Duke University Medical Center, Durham, North Carolina

**Keywords:** osteomyelitis, lateral corpectomy, retroperitoneal transpsoas, minimally invasive, spine surgery

## Abstract

In this video, the authors highlight the operative treatment of a 55-year-old man with chronic osteomyelitis discitis. The operation entailed a minimally invasive lateral retroperitoneal transpsoas approach for L3 and L4 corpectomies, L2–5 interbody fusion, and L2–5 minimally invasive posterior instrumentation. The operation proceeded in two stages, beginning in the lateral position with corpectomy of the L3 and L4 vertebral bodies and placement of a corpectomy cage. After closure of this access wound, the patient was turned to a prone position for the posterior element of the operation. Posterior instrumentation was placed with pedicle screws at L2 and L5.

The video can be found here: https://stream.cadmore.media/r10.3171/2022.3.FOCVID2210

## Transcript

### 0:21 Patient Introduction.

A 55-year-old man who had undergone previous L1–4 laminectomies and a full course of antibiotics for lumbar osteomyelitis presented with continued low-back pain and right leg pain affecting his groin and thigh, as well as gait instability. His symptoms were exacerbated with physical activity and alleviated by elevating his right leg. On examination, he was neurologically intact with full strength, intact sensation to light touch, and preserved gait. His standing scoliosis films and CT imaging demonstrated continued osteomyelitis discitis at L3–4 with significant destruction of these vertebral bodies and progressive kyphotic deformity.^1^

### 0:58 Operative Plan.

Based on his symptoms and imaging findings, we offered the patient a minimally invasive lateral retroperitoneal transpsoas approach for L3 and L4 corpectomies, L2–5 interbody fusion, and L2–5 minimally invasive posterior instrumentation.^2–4^ Standard operative risks to nearby neural, bowel, and vascular structures applied, and alternative options included anterior corpectomy, posterior fusion without corpectomy, or no surgery.^5^ L2–5 posterior instrumentation was chosen to safely minimize the construct length and biomechanical disruption. Critical structures to avoid during the lateral access include the major vasculature and lumbar plexus as the exiting nerve roots course within and around the psoas muscle in the retroperitoneal space. This is aided with use of neuromonitoring throughout the case. Informed written consent was obtained from the patient who wished to proceed.

### 1:48 Room Setup and Positioning for Lateral Stage.

The patient was brought to the operating room where a formal timeout was performed verifying the correct patient, procedure, and side. General anesthesia was induced and the patient was endotracheally intubated. Neuromonitoring needles were placed in the bilateral lower extremities for electromyography monitoring, motor evoked potentials, and somatosensory evoked potentials. For stage 1 of the procedure, he was positioned in the right lateral decubitus position. An axillary roll was placed. His arms were kept outstretched on the appropriate arm boards. He was taped securely to the table with his legs appropriately padded. The table was flexed so as to open up the space between his left 12th rib and iliac crest. Fluoroscopy was used to position the table so that true AP and lateral images of the spine would be obtained. His L2–3 and L4–5 disc spaces were marked on the skin. An incision was planned spanning from disc space to disc space. This planned incision was prepped and draped in the usual sterile fashion; local anesthetic was administered.

### 2:44 Initial Incision and Retractor Placement.

Incision was made with a no. 10 blade scalpel, and deeper dissection down to the muscular fascia performed with Bovie cauterization. Once we encountered muscular fascia, it was incised with a scalpel. Blunt dissection was performed through the musculature down to the level of the spine. An initial dilator was docked on the L4–5 disc space, and a K-wire was introduced into the disc space. Circumferential stimulation of the initial dilator and the two subsequent demonstrated no nearby neuromonitoring signal.^6^ An expandable retractor was placed and a pass probe stimulator used to ensure there were no nearby nerve structures. In this case, the iliac crest was not obstructive to the approach. However, patients with a high iliac crest can present a challenge with accessing the L4–5 disc. Breaking the bed to extend the space between the lower rib and iliac crest can improve accessibility, as well as slight angling of the retractor and using angled instruments. Additionally, verifying aorta and IVC locations anterior to the vertebral body on preoperative imaging is critical to the safety of this operation, along with maintenance of a posterior approach during the initial access stage. The vessels were not directly visualized in this case.

### 3:54 Discectomies and Corpectomies.

The disc space was bipolar coagulated and incised with a scalpel. Using a variety of disc preparation instruments, including a Cobb elevator, curettes, curved curettes, ringed curettes, and rasps, a thorough L4–5 discectomy was performed. The discectomy process was then repeated at L2–3. The dilators were then docked on the center of the L3–4 bony mass. Circumferential stimulation did not demonstrate any nearby neuromonitoring signal, and residual psoas muscle on the vertebral bodies was bipolar coagulated and elevated off the bone. Then, using osteotomes, L3 and L4 corpectomies were performed by removing the bone piecemeal. Any bony bleeding can be controlled with hemostatic agent and packing, and generally will cease upon completion of the corpectomy. The ALL was left intact.

For this stage, initial completion of the upper and lower discectomies is critical to providing reference points for the extent of the operative field. Alternative techniques can include the use of a diamond drill, which improves working hemostasis; however, the osteotome is typically faster. Intraoperative x-rays provide feedback on the extent of resection and verification of completion when the instrument tips reach the deep portion of the contralateral annulus, as shown here.

### 5:04 Placement of Expandable Cage and Lateral Wound Closure.

Calipers were used to determine the appropriate sized cage. This titanium expandable cage was fit with 12° endplates on either end. The cage was packed with bone morphogenetic protein and morselized allograft and tamped into the corpectomy defect. Once it had good experience on AP fluoroscopy, the fluoroscopy machine was turned to the lateral position and the cage was expanded until it was of a snug fit and good appearance on fluoroscopy. The locking mechanism was engaged and the corpectomy defect packed with more morselized allograft. This completed the L2–5 interbody fusion. The wound was copiously irrigated and closed in layers with 0 Vicryl sutures in the muscular fascia, 2-0 in the subcutaneous fat, 3-0 in the dermis, and staples in the skin. This concluded stage 1 of the procedure.

### 5:52 Positioning and Incisions for Posterior Stage.

For stage 2 of the procedure, the patient was position prone on the Jackson table compatible with the intraoperative CT scanner. Care was taken to pad all pressure points, including his wrists, elbows, iliac crest, thighs, knees, and feet. His back was prepped and draped in the usual sterile fashion, and AP fluoroscopy was used to mark the bilateral location of his L2 and L5 pedicles. At each of these locations, a skin incision was made with a scalpel and deeper dissection through the lumbar muscular fascia performed with Bovie cauterization.

### 6:21 Posterior Instrumentation.

A Jamshidi needle was cannulated through each pedicle coursing from lateral to medial using AP fluoroscopy. Once the medial aspect of the pedicle was encountered by each Jamshidi needle, the fluoroscopy machine was turned to the lateral position and the Jamshidi needles were tamped the rest of the way into the vertebral bodies. An undersized tap was used to prepare each screw tract over the K-wire and then appropriately sized pedicle screws were placed over the wire. All screws had good purchase. At this point, we performed an intraoperative CT scan. This demonstrated perfect placement of his instrumentation and significant improvement in his lumbar lordosis. Calipers were used to determine the appropriate length rods. These rods were passed subfascially through the screw extension tabs and set screws were placed. After confirming good appearance on final AP and lateral fluoroscopy, the set screws were final tightened with the extension tabs broken off.

### 7:22 Posterior Wound Closure and Completion of Procedure.

The wounds were copiously irrigated. They were each closed with 0 Vicryl sutures in the fascia, 2-0 Vicryl sutures in the subcutaneous fat, 3-0 in the dermis, and staples in the skin. The wounds were dressed sterilely and the patient returned supine to his stretcher. He tolerated the procedure without apparent complication.

### 7:41 Postoperative Course.

At his most recent follow-up 12 months postoperatively, the patient reported 95% improvement in his preoperative symptoms with some back pain but no leg pain. He had full strength on examination with no evidence of instrumentation failure on postoperative films.

## Disclosures

Dr. Erickson reported other from Medtronic, Globus, and DePuy Synthes, outside the submitted work. Dr. Abd-El-Barr reported other from Spineology, DePuy Synthes, and Trackx, outside the submitted work. Dr. Shaffrey reported personal fees from NuVasive, Medtronic, Zimmer Biomet, SI Bone, and Proprio, outside the submitted work. Dr. Than reported personal fees from Bioventus, DePuy Synthes, Accelus, and Globus, outside the submitted work.

## Author Contributions

Primary surgeon: Than. Assistant surgeon: Wang. Editing and drafting the video and abstract: Srinivasan, Wang, Abd-El-Barr, Shaffrey, Than. Critically revising the Work: Srinivasan, Wang, Abd-El-Barr, Shaffrey, Than. Reviewed submitted version of the work: all authors. Approved the final version of the work on behalf of all authors: Srinivasan. Supervision: Shaffrey, Than. Nurse practitioner working with the surgeon: Rapoport.

## Supplemental Information

### Patient Informed Consent

The necessary patient informed consent was obtained in this study.
